# A novel computational framework for deducing muscle synergies from experimental joint moments

**DOI:** 10.3389/fncom.2014.00153

**Published:** 2014-12-03

**Authors:** Anantharaman Gopalakrishnan, Luca Modenese, Andrew T. M. Phillips

**Affiliations:** ^1^The Royal British Legion Centre for Blast Injury Studies at Imperial College LondonLondon, UK; ^2^Structural Biomechanics, Department of Civil and Environmental Engineering, Imperial College LondonLondon, UK; ^3^Griffith Health Institute, Centre for Musculoskeletal Research, Griffith UniversityGold Coast, QLD, Australia

**Keywords:** muscle synergies, musculoskeletal modeling, healthy gait, joint moments, direct collocation, non-negative matrix factorization, effort minimization

## Abstract

Prior experimental studies have hypothesized the existence of a “muscle synergy” based control scheme for producing limb movements and locomotion in vertebrates. Such synergies have been suggested to consist of fixed muscle grouping schemes with the co-activation of all muscles in a synergy resulting in limb movement. Quantitative representations of these groupings (termed muscle weightings) and their control signals (termed synergy controls) have traditionally been derived by the factorization of experimentally measured EMG. This study presents a novel approach for deducing these weightings and controls from inverse dynamic joint moments that are computed from an alternative set of experimental measurements—movement kinematics and kinetics. This technique was applied to joint moments for healthy human walking at 0.7 and 1.7 m/s, and two sets of “simulated” synergies were computed based on two different criteria (1) synergies were required to minimize errors between experimental and simulated joint moments in a musculoskeletal model (pure-synergy solution) (2) along with minimizing joint moment errors, synergies also minimized muscle activation levels (optimal-synergy solution). On comparing the two solutions, it was observed that the introduction of optimality requirements (optimal-synergy) to a control strategy solely aimed at reproducing the joint moments (pure-synergy) did not necessitate major changes in the muscle grouping within synergies or the temporal profiles of synergy control signals. Synergies from both the simulated solutions exhibited many similarities to EMG derived synergies from a previously published study, thus implying that the analysis of the two different types of experimental data reveals similar, underlying synergy structures.

## Introduction

**Figure 1:**
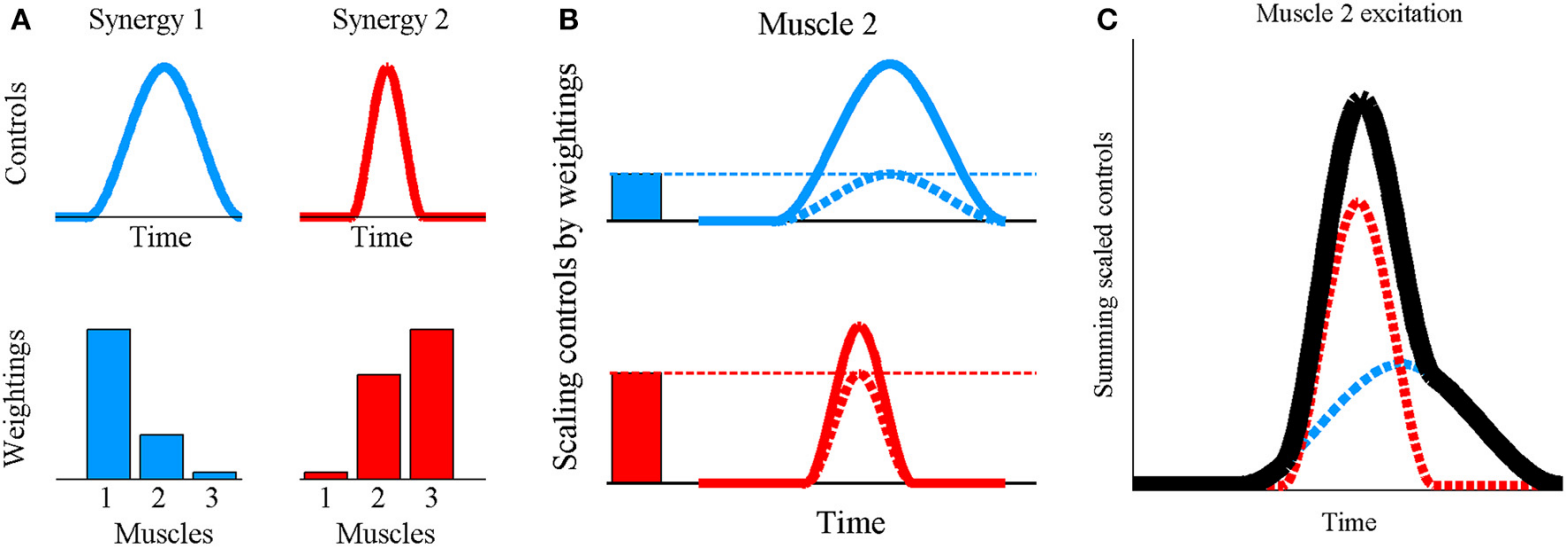
**Synergy combination principle for synchronous synergies where (A) the invariant grouping of muscles within the synergy is quantitatively represented by a set of scalar muscle weightings and the co-activation of muscles belonging to a synergy depends on a time-varying control signal. (B)** To obtain the excitation profile for muscle 2, each synergy's control signal (continuous lines) is scaled by the weighting for muscle 2 in that synergy (solid bars) obtaining the scaled synergy controls (dashed lines) **(C)** followed by algebraic summation of the scaled signals (dashed lines) to obtain the muscle excitation (black line). In essence, this is a linear combination process defined by u = ∑W.h where u is a vector of muscle excitations (0 ≤ *u* ≤ 1), W is a vector of muscle weightings and h is the scalar, instantaneous value of the synergy's control signal. The summation is taken over all synergies.

The human musculoskeletal system is characterized by muscle redundancy, where the muscles outnumber the degrees of freedom which they control thus resulting in infinite solutions to the load sharing problem (Modenese et al., 2013). While constraints on individual muscle forces due to fatigue (Singh et al., 2010), force-length relationships (John et al., 2013), and inter-muscular force coupling during multi-joint movements (Zajac and Gordon, 1989) may represent some of the multiple factors that highlight the need for redundant musculature, it nevertheless complicates the search for a general control principle underlying the muscle recruitment patterns observed for various tasks. The muscle synergy hypothesis describes one such potential control architecture governing limb movements in vertebrates, and is supported by results from several detailed experimental studies on animals (Giszter et al., 1993; Jacobs and Macpherson, 1996; Tresch et al., 1999; d'Avella et al., 2003; Ting and Macpherson, 2005). According to this hypothesis, muscle excitation signals are not individually regulated, but muscles are organized at the spinal level into groups known as synergies, with muscles in the same synergy being simultaneously activated. In this study, a synergy is defined by a time-varying control signal and a set of muscle-specific scalar constants known as the muscle weightings (Ting and Macpherson, 2005)—collectively termed the “synergy structure.” The excitation signal reaching a muscle is formed by scaling each synergy's control signal by the muscle's weighting value in that synergy, followed by algebraic summation of the scaled signals from all synergies (Ting and Macpherson, 2005)—a process referred to as “synergy combination” (Figure 1). This study hypothesizes a previously investigated, potential controller layout (Ting and Macpherson, 2005; Clark et al., 2010; Chvatal and Ting, 2013) where muscle weightings are stored at the spinal cord level as invariant patterns, while the time-varying synergy controls are modulated according to the desired movement, to then undergo synergy combination and produce a range of limb movements. Since changes to a synergy's muscle weightings would not alter the temporal profile of the synergy's contributions toward muscle excitations, weightings are considered to embody “spatial” synergy characteristics while the control signals are associated with the “temporal” characteristics.

Experimental studies have traditionally used methods such as non-negative matrix factorization (NNMF) (Lee and Seung, 1999), factor analysis (Ivanenko et al., 2006), and independent component analysis (Tresch et al., 2006) to factorize experimental electromyographic signals (EMGs) (taken to be representative of muscle excitations) and obtain quantitative representations for muscle weightings and synergy controls. A perceived advantage of having a synergy based controller is the ability to vary only one of either spatial (muscle weightings) or temporal (synergy controls) aspects of the synergy structure to produce a wide range of movements (d'Avella and Bizzi, 2005) and hence factorization is usually applied to EMG recorded for a family of functionally similar movements—e.g., jumping, swimming, and kicking in frogs (d'Avella and Bizzi, 2005). The computed synergies are required to explain variations in EMG observed across different movements in the family (inter-task variation) and between repetitions of the same movement (intra-task variation). However, a good level of expertise at EMG measurement is needed to achieve accurate electrode placement (Mesin et al., 2009), ensure the avoidance of cross-talk between adjacent muscles (De Luca, 1997; Disselhorst-Klug et al., 2009) and utilize indwelling EMG electrodes to record the activity in deeper muscles (Perotto et al., 2011). The qualitative nature of EMG also limits the nature of the inferences that can be drawn from EMG derived synergies, which are further constrained by the practical limitations of obtaining EMG from all the muscles involved in a movement (De Rugy et al., 2013; Steele et al., 2013). This has warranted a search for alternative techniques to deduce the structure of muscle synergies.

As inter and intra-task variations in kinematics (e.g., joint angles, end-effector coordinates) and kinetics (e.g., joint moments, end-effector forces) of a movement task must also be consistent with an underlying synergy based controller, prior studies have utilized musculoskeletal simulations to compute muscle synergies that are consistent with such variations (Moghadam et al., 2011; Kutch and Valero-Cuevas, 2012; McKay and Ting, 2012; Steele et al., 2013; De Groote et al., 2014). Such studies commonly compute synergies that can generate a family of forces in a static kinematic configuration or analyze dynamic movement tasks on a frame by frame basis using static analysis techniques (a “quasi-static” approximation). Such approaches have computed synergies from isometric joint moments (Moghadam et al., 2011) and isometric end-effector forces (Kutch and Valero-Cuevas, 2012; Borzelli et al., 2013; Steele et al., 2013), by first estimating muscle activations which reproduced the target kinetics in a musculoskeletal model followed by factorization of the simulated activations to obtain muscle synergies. When muscle redundancy implies the existence of multiple, equally feasible solutions for the activation patterns, an effort minimization criteria is commonly used to identify a unique solution, such as in the study by De Groote et al. (2014). This study used static optimization (Crowninshield and Brand, 1981) to compute muscle activations for walking (quasi-static approximation), followed by NNMF of activations to compute the muscle synergy structure. While these methods succeed in identifying synergy groupings for all muscles in the model that are involved in a task, they cannot dissociate the concepts of synergies and optimality during the computation process. Static methods would also be unsuitable for dynamic movement tasks where synergy structure could be influenced by the non-linear, history-dependent musculotendon behavior which is normally modeled by differential equations. Experimental evidence has also suggested that muscle synergies may be modulated by afferent sensory feedback (Cheung et al., 2005). Afferent feedback from structures such as muscle spindles and Golgi tendon organs are usually modeled as differential equations in time (Mileusnic and Loeb, 2006; Mileusnic et al., 2006; Kistemaker et al., 2013), and the effects of sensory feedback on synergy structure cannot be identified from a simulation technique that is incapable of accounting for dynamic effects.

While studies have previously used EMG derived muscle synergies to inform dynamic simulations of movement (Neptune et al., 2009; McGowan et al., 2010; Allen and Neptune, 2012; Sartori et al., 2013; Walter et al., 2014), the deduction of muscle synergies from the kinematics and kinetics of dynamic movement tasks remains largely undeveloped. A recent study by Ruckert and d'Avella (2013) computed synergies for walking and reaching tasks in a dynamic model by defining a family of movements using sets of basic kinematic goals—e.g., varying stride lengths, reaching target locations—and applying a reinforcement learning algorithm. However, during impaired movement the task goals might be unknown and even highly subject-specific, thus complicating the reproduction of a specific subject's experimental movement characteristics in the model. Therefore, this study began with the hypothesis that experimentally recorded kinematics and kinetics could be used to infer muscle synergy structure for a family of movements and formulated a methodology—termed “synergy constrained torque decomposition”—for achieving the same. The method incorporated dynamic models of the neuromuscular system, deduced muscle synergies of quantitative value (see Discussion) and was capable of assigning synergies to all muscles within the system—in this study, a musculoskeletal model of the lower limb—thus extending the capabilities of current synergy computation techniques.

Variations in both experimental kinematics as well as kinetics are reflected in joint moments computed using the inverse dynamics methodology. In this study, a subject's “biomechanical output” was hence taken to be adequately quantified by their joint moments, and synergy constrained torque decomposition sought to decompose joint moments for a family of movements into muscle synergies. This study focused on the computation of muscle synergies controlling healthy human gait and a family of movements was defined as gait at slow and fast walking speeds. The feasibility of two criteria which the computed synergies were required to satisfy was tested: (1) minimize errors between experimental and simulated joint moments in the model (termed the pure-synergy solution), (2) in addition to joint moment errors, also minimize muscle effort (optimal-synergy solution). The pure-synergy solution represented a control strategy aimed at tracking experimental joint moment profiles as accurately as possible. A comparison of pure-synergy and optimal-synergy solutions was expected to yield insights into how the structure of a fixed number of synergies could be altered to reduce muscle effort, without substantially changing the biomechanical output. Muscle activation and contraction dynamics were included in the torque decomposition problem forming an optimal control problem (OCP) that was solved using direct collocation (Kaplan and Heegaard, 2001). Finally, the similarity of the synergies computed on the basis of the two criteria to EMG derived synergies (reported by Clark et al., 2010) was quantitatively assessed. This comparison was intended to provide insight on whether synergies derived from different types of experimental data exhibited similarities in structure.

## Methods

### Movement data

A single male subject (24 years, height 174 cm, weight 72 kg) walked on an instrumented treadmill (Bertec, Columbus, OH) at slow, intermediate and fast speeds of 0.7, 1.3, and 1.7 m/s, respectively. 3D marker-based motion capture (Motion Analysis, Santa Rosa, CA) recorded the subject's kinematics at 60 Hz while a force plate embedded in the treadmill synchronously measured tri-axial ground reaction forces and moments at 2100 Hz. Informed consent was obtained from the subject and the protocol was certified by the Internal Review Board of the University of Delaware where the motion capture data was collected. Muscle synergies were deduced from the kinematics and kinetics of three consecutive, arbitrarily chosen gait cycles at the slow and fast walking speeds. Data from three similarly chosen gait cycles at the intermediate speed was used to validate the “biomechanical relevance”—the ability to reproduce experimental joint moments—of the computed synergies (described in Section “Simulations”). A gait cycle was defined as the time interval between two consecutive heel strikes of the right foot.

### Musculoskeletal model

**Figure 2:**
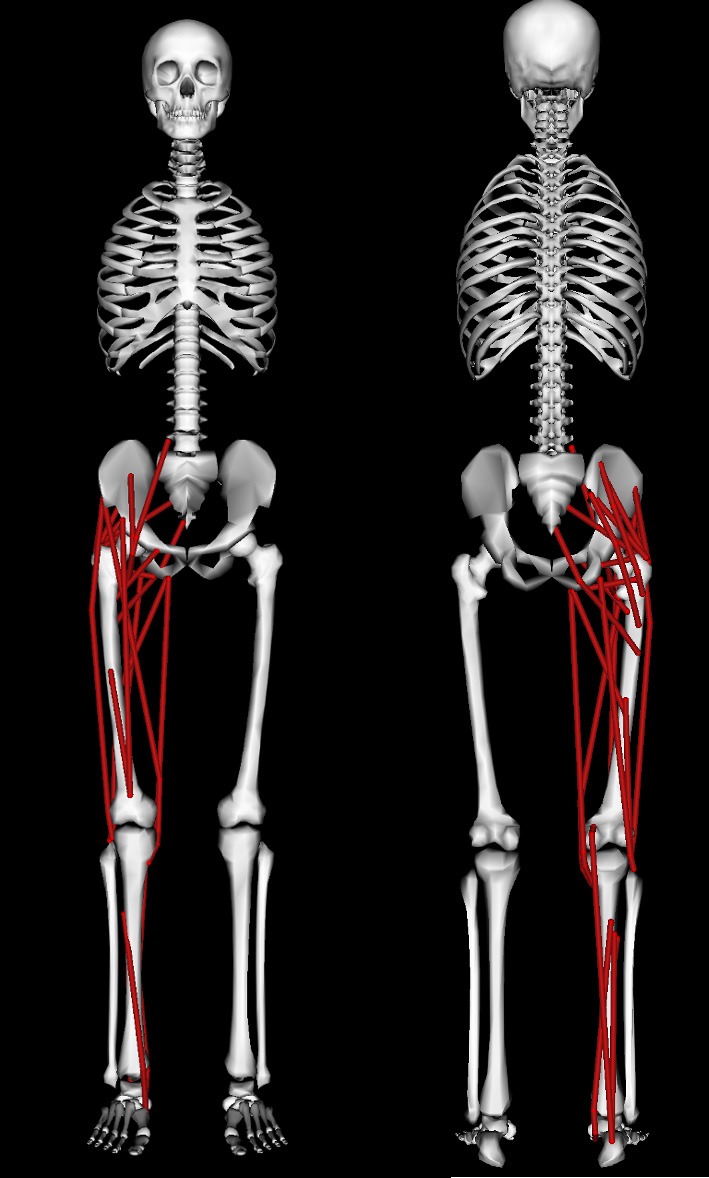
**Anterior (left) and posterior (right) views of the musculoskeletal model used in this study**. The model is described in detail in the Section on Methods.

A modified version of the 3D “gait2354” musculoskeletal model (Delp et al., 1990; Anderson and Pandy, 1999) distributed with the OpenSim (Delp et al., 2007) software package (available for download at https://simtk.org/home/opensim) was used in this study (Figure 2). This whole body model possessed 23 mechanical DOFs and 54 muscles, of which 24 muscles spanning the right leg were retained (see Table 1) to enable comparisons with EMG derived synergies reported for a single leg (Clark et al., 2010). Toe and subtalar DOFs were locked on both legs leaving 19 DOFs. Torso and left leg DOFs were retained in the model to account for their coupling effects on the right leg joint moments and were actuated by torques that were directly applied to the DOF. Based on the recommendations of Arnold et al. (2013), the tendon strain at which its force equaled the muscle's maximum isometric force (the parameter labeled “FmaxTendonStrain” in the OpenSim model) was set to 4% for all muscles except the plantarflexors which were set to 10% to account for their higher tendon compliances observed from ultrasound measurements (Farris and Sawicki, 2012). Experimental marker data was used in OpenSim to scale the model to match the subject's dimensions followed by inverse kinematics (Lu and O'Connor, 1999) to obtain joint angle kinematics for walking. The details of OpenSim's scaling and inverse kinematics tools are described in the study by Delp et al. (2007). Muscle-tendon lengths and moment arms of all muscles over the three chosen gait cycles at each speed were computed for later use during synergy constrained torque decomposition and subsequent validation. OpenSim's inverse dynamics tool was then used to compute experimental joint moments from joint kinematics and experimental ground reaction force data.

**Table 1:** **Names and abbreviations of all muscles on the right leg of the musculoskeletal model**.

**Muscle**	**Abbreviation**
Gluteus medius (Element 1)	GMd1
Gluteus medius (Element 2)	GMd2
Gluteus medius (Element 3)	GMd3
Long head of the biceps femoris	LH
Short head of the biceps femoris	SH
Sartorius	SR
Adductor magnus	AM
Tensor fascia latae	TF
Pectineus	PC
Gracilis	GR
Gluteus maximus (Element 1)	GMx1
Gluteus maximus (Element 2)	GMx2
Gluteus maximus (Element 3)	GMx3
Iliacus	IL
Psoas major	PM
Quadriceps femoris	QF
Gemelli	GE
Piriformis	PI
Rectus femoris	RF
Vastus intermedius	VI
Medial gastrocnemius	MG
Soleus	SO
Tibialis posterior	TP
Tibialis anterior	TA

### Synergy constrained torque decomposition: joint moments to muscle synergies

In the hypothesized synergy based controller, muscle weightings are expected to be invariant across walking speeds while the time-varying synergy control patterns may vary. In the torque decomposition problem, muscle excitations from synergy combination of those weightings and controls were expected to produce experimental joint moments (over all gait cycles at slow and fast speeds) in the musculoskeletal model, when used as inputs to models of muscle activation and contraction dynamics. The principle of reproducing joint moments using muscle dynamic models alone was similar to the hybrid forward-inverse model proposed by Buchanan et al. (2005) since joint moments, muscle-tendon lengths and moment arms were pre-computed, thus excluding the computationally expensive skeletal dynamics (equations of motion) from the problem definition. For both slow and fast walking speeds, torque decomposition was performed over the three selected gait cycles of experimental data. Joint moments at 5 DOFs in the right leg (flexion/extension, adduction/abduction, and internal/external rotation at the hip, extension/flexion at the knee and dorsiflexion/plantarflexion at the ankle) were decomposed into muscle synergies controlling the 24 right leg muscles. The following OCP defined the torque decomposition problem.

(1A) $$\begin{array}{l} \underset{W_{ik},h_{sk}}{\text{minimize}}\;J=\sum_{s\;=\;1}^{2}\int_{0}^{T_{s}^{3}}\sum_{j\;=\;1}^{N_{J}}{\left[\left(M_{j}\left(t\right)\;-M_{j}^{*}\left(t\right)\right)\right]}^{2}.dt\\ \;k=1,2\ldots N_{syn} \end{array}$$

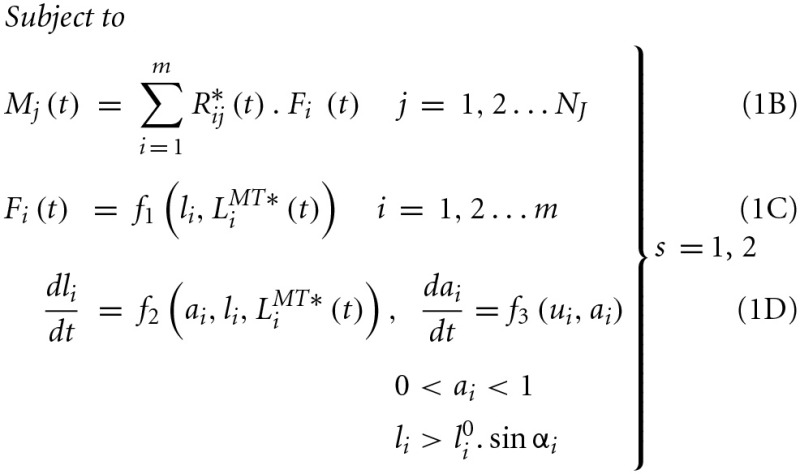

(1E) $$\begin{array}{l} u_{is}\left(t\right)\;=\sum_{k\;=\;1}^{N_{syn}}W_{ik}\;.\;h_{sk}\left(t\right)\;i=1,2\ldots m\\ \;0<u_{is}<1 \end{array}$$

Table 2 provides a detailed description of the terms in Equation (1). However, to summarize, Equation (1A) is the cost function for minimizing the squared errors between experimental and simulated joint moments. Equation (1B) describes the mapping from individual muscle forces to joint moments at each DOF, while Equations (1C,D) collectively represent the muscle's dynamic behavior. The function *f*_1_ represents the computation of muscle force from fiber length while functions *f*_2_ and *f*_3_ in Equation (1D) represent muscle contraction (force-length and force velocity relationships) and activation dynamics, respectively. The functions *f*_1_ and *f*_2_ were based on the equations from Thelen (2003) while *f*_3_ used the equations described by He et al. (1991). Since *a*_*i*_, *l*_*i*_, and *u*_*i*_ were functions of time and their values differed between walking speeds, Equations (1B–D) had to be independently enforced for each of the *s* walking speeds as implied by the large bracket (the *s* index has been left out from these equations in the interest of readability). Finally, Equation (1E) is a linear synergy combination model, denoting the relationship between muscle excitations and synergy weightings and controls. Values of *W*_*ik*_ and *h*_*sk*_ formed the ultimate solution to the OCP.

**Table 2:** **Description of terms in Equations (1A–E)**.

**Equation (1)**	**Description of terms**
(A)	*s* – Slow (*s* = 1) and fast (*s* = 2) walking speeds
	*T*^3^_*s*_ – Duration of 3 gait cycles at the *s*^*th*^ walking speed
	*M*_*j*_ – Simulated joint moment at *j*^*th*^ DOF
	M^*^_j_ – Experimental joint moment at *j*^*th*^ DOF
	*N*_*J*_ – Total number of DOFs (*N*_*J*_ = 5)
	*t* – Time
	*W*_*ik*_ – Muscle weighting of the *i*^*th*^ muscle in the *k*^*th*^ synergy
	*h*_*sk*_ – Synergy control for the *k*^*th*^ synergy at the *s*^*th*^ walking speed
	*N*_*syn*_ – Total number of synergies
(B)	R^*^_ij_ – Experimental moment arm of the *i*^*th*^ muscle about *j*^*th*^ DOF
	*F*_*i*_ – Force in the *i*^*th*^ muscle
	*m* – Total number of muscles (*m* = 24)
(C)	*f*_1_ – Function computing muscle force from fiber length and muscle-tendon length
	*l*_*i*_ – Fiber length of the *i*^*th*^ muscle
	*L*^MT*^_i_ – Experimental muscle-tendon lengths of the *i*^*th*^ muscle
(D)	*f*_2_ – Muscle contraction dynamics
	*f*_3_ – Muscle activation dynamics
	*a*_*i*_ – Activation level in the *i*^*th*^ muscle
	*u*_*i*_ – Excitation reaching the *i*^*th*^ muscle
	*l*^0^_*i*_,α_*i*_ – Optimal fiber length and pennation angle at optimal fiber length, of the *i*^*th*^ muscle
(E)	*u*_*is*_ – Excitation in the *i*^*th*^ muscle during the *s*^*th*^ trial (For a description of other terms, see Equation 1A)

The solution to this highly non-linear OCP was pursued using the numerical method of direct collocation (Kaplan and Heegaard, 2001; Ackermann and Van Den Bogert, 2010; Kistemaker et al., 2013). Direct collocation computes the values of the model's state variables (*a*_*i*_ and *l*_*i*_), control inputs (*h*_*sk*_) and invariant control parameters (*W*_*ik*_) at *P*_*s*_ discrete equispaced points of time (known as collocation points) instead of computing them as continuous functions of time as with an analytical approach. This discretization process permits the OCP to be transformed into a non-linear optimization problem which was then solved using the gradient based SNOPT algorithm (Gill et al., 2002). Having 120 and 90 collocation points (points were roughly 0.027 s apart at both slow and fast walking speeds) cumulatively over the three gait cycles of the slow and fast walking trials respectively was sufficient to yield a solution that could be reproduced by a forward dynamic simulation of the system's governing Equations (1B–E). This was important to ensure that the OCP solution adhered to system dynamics despite approximations arising from temporal discretization during the solution process. During direct collocation, the lower bounds on *a*_*i*_ and *u*_*i*_ were set to a nominally low value of 0.01 (Thelen and Anderson, 2006) instead of 0 in order to avoid singularities in the muscle model. The initial guess for direct collocation was obtained by using arbitrary values for *W*_*ik*_, *h*_*sk*_ which were in turn computed by the NNMF of arbitrary muscle excitation patterns (constant values or sine function pulses). The procedure for deriving the arbitrary initial guesses is detailed in the Appendix. The excitation patterns and initial muscle state values were used to integrate the muscle dynamic equations over all gait cycles of interest at both speeds. The values of *W*_*ik*_ along with temporally discretized values of *h*_*sk*_ and the integrated muscle states at each collocation point constituted the initial guess for direct collocation. Torque decomposition was implemented in MATLAB (2013a, Mathworks, Natick MA).

### Simulations

Two variants of the synergy constrained torque decomposition problem were solved:

1. **Pure-synergy solution**: The original OCP (Equations 1A–E) was solved to compute muscle weightings (*W*_*ik*_) and synergy controls (*h*_*sk*_). As four to five EMG derived lower limb synergies have been observed for locomotion tasks (Ivanenko et al., 2004; Cappellini et al., 2006; Clark et al., 2010), five pure-synergy solutions were computed by varying the number of synergies (*N*_*syn*_) between three and seven. For each value of *N*_*syn*_, synergy constrained torque decomposition was repeated with 15 different initial guesses and the solution yielding the minimum value of *NRMSE* was selected (see Appendix). The goodness of joint moment reproduction in the model was quantified by a normalized root mean squared value of the joint moment errors (referred to as *NRMSE*) shown in Equation (2) which was based on Sartori et al. (2013). The maximum and minimum values of joint moment in the denominator were taken cumulatively over all speeds, collocation points and DOFs.
  (2) $$\begin{array}{l} NRMSE=\frac{1}{N_{J}.\sum_{s\;=\;1}^{2}P_{s}}\\ \;\sqrt{\frac{1}{{\left(\text{max}M_{jn}^{*}-\text{min}M_{jn}^{*}\right)}^{2}}\sum_{s\;=\;1}^{2}\sum_{n\;=\;1}^{P_{s}}\sum_{j\;=\;1}^{N_{J}}{\left(M_{jn}-M_{jn}^{*}\right)}^{2}\;} \end{array}$$
  To determine a conservative, minimum value of *N*_*syn*_ needed to accurately simulate walking joint moments in the model (1) the corresponding joint moment errors had to be low but (2) not too low to permit the existence of biomechanically redundant synergies which only made very small improvements to the joint moment errors. These two requirements were respectively quantified by upper and lower limits on the *NRMSE* value which had to necessarily be satisfied by the pure-synergy solution corresponding to the minimum *N*_*syn*_ value. The upper limit defined the *NRMSE* below which joint moment reproduction could be considered low, and was determined by setting (*M*_*jn*_ − M^*^_jn_) in Equation (2) to 5% of the difference between maximum and minimum joint moments at the respective DOFs and walking speeds. The lower limit on the *NRMSE* was computed by setting (*M*_*jn*_ − M^*^_jn_) in Equation (2) to a very small value of 1 Nm at all DOFs, speeds and collocation points. Since the *NRMSE* was expected to decrease with increasing values of *N*_*syn*_, the highest value of *N*_*syn*_ for which *NRMSE* lay within the upper and lower limits was chosen to be the minimum number of synergies needed.
  Since the hypothesized synergy structure required invariance of muscle weightings across walking speeds, the biomechanical relevance of the minimum *N*_*syn*_ value and corresponding synergy structure was further tested. In this procedure, muscle weighting values *W*_*ik*_ deduced from synergy constrained torque decomposition of slow and fast walking data were considered constants in Equation (1E) while the cost function in Equation (1A) was modified to minimize joint moment errors over three gait cycles at the intermediate walking speed (1.3 m/s). The OCP could thus only vary control signal patterns *h*_*sk*_ to minimize the joint moment errors.
2. **Optimal-synergy solution:** The cost function of the original OCP was appended with an additional term aimed at minimizing the sum of muscle activation squares over all time instants (Ackermann and Van Den Bogert, 2010). The temporally discretized version of the cost function for direct collocation is presented in Equation (3) where *M*_*jn*_ and *a*_*jn*_ denote joint moments (*j*^*th*^ DOF) and muscle activations (*j*^*th*^ muscle) at the *n*^*th*^ collocation point. The number of synergies *N*_*syn*_ was set to the minimum value identified earlier from the pure-synergy solutions.
  The scalar weighting constant *K* in Equation (3) was determined by iteratively repeating the optimal-synergy simulation with increasing values of *K* ranging from 0 (pure-synergy solution) to 500 in steps of 100. With increasing values of *K*, the activation cost (coefficient of *K* in Equation 3) of the corresponding optimal-synergy solution would be expected to decrease due to greater penalization of that term within the cost function. Thus, beyond a certain threshold *K*-value, the reduction in activation cost may be insignificant and also unnecessarily prioritizing the minimization of activation cost over joint moment errors. To quantify the minimum desired reduction in activation cost between the solutions for *K* and *K* + 100 (Δ_*K*:*K*+100_), Equation (4) computed the magnitude of reduction when activations at each collocation point in the *K* solution (*a*^*K*^_*jn*_) were hypothetically lowered by a very small value (0.01, the lower bound on muscle activation). If the actual change in activation cost between *K* and *K* + 100 was lower than Δ_*K*:*K*+100_, the improvement was considered insignificant and the solution corresponding to *K* was chosen to be final.
  (3) $$J=\sum_{s\;=\;1}^{2}\sum_{n\;=\;1}^{P_{s}}\left(\sum_{j\;=\;1}^{N_{J}}{\left[M_{jn}\;-M_{jn}^{*}\right]}^{2}+K\;.\;\sum_{j=1}^{m}a_{jn^{2}}\right)$$
  (4) $$\begin{array}{l} {△}_{K:K+100}\;=\sum_{s\;=\;1}^{2}\sum_{n\;=\;1}^{P_{s}}\sum_{j\;=\;1}^{N_{J}}({\left(a_{jn}^{K}\right)}^{2}\\ \;-\;{\left(max\left(0.01,a_{jn}^{K}\;-\;0.01\right)\right)}^{2}) \end{array}$$

The following analyses were then performed:

- A synergy-specific similarity index (SI) was defined as the dot-product of the normalized muscle weightings (unitary value of this index means identical muscle grouping in synergies). Muscle weightings from the pure-synergy and optimal-synergy solutions were compared using this index. A mean similarity index was computed by averaging these indices over all synergies. Similarity indices greater than 0.8 were considered to be indicative of a high degree of similarity between synergies, while values between 0.5 and 0.8 and lesser than 0.5 were considered moderate and low, respectively. Control signals from every synergy, at each of the slow and fast speeds were individually compared by computing their Pearson's correlation coefficient (ρ). An overall comparison of synergy controls from the two solutions was achieved through a “mean correlation coefficient” for which control signals from each synergy and walking speed (interpolated at 30 equally spaced time-points over a gait cycle) into a single vector and the Pearson's correlation coefficient between vectors corresponding to either solution was computed. Muscle activations derived from the two solutions were also visually studied. High, moderate, and low correlations were classified on the same ranges prescribed for the similarity index.
- Muscle weightings and synergy controls from the pure-synergy and optimal-synergy solutions were then compared to those obtained from NNMF of experimental EMG and reported in a study by Clark et al. (2010). Correspondent synergies were first identified by using the previously defined similarity index following which synergy controls were compared by computing both individual and mean correlation coefficients. Comparisons of muscle weightings were restricted to those muscles for which EMG was recorded in the study. Synergy controls from Clark et al. for speeds of 0.6 and 1.6 m/s were compared to those at 0.7 and 1.7 m/s in our study.

## Results

**Figure 3:**
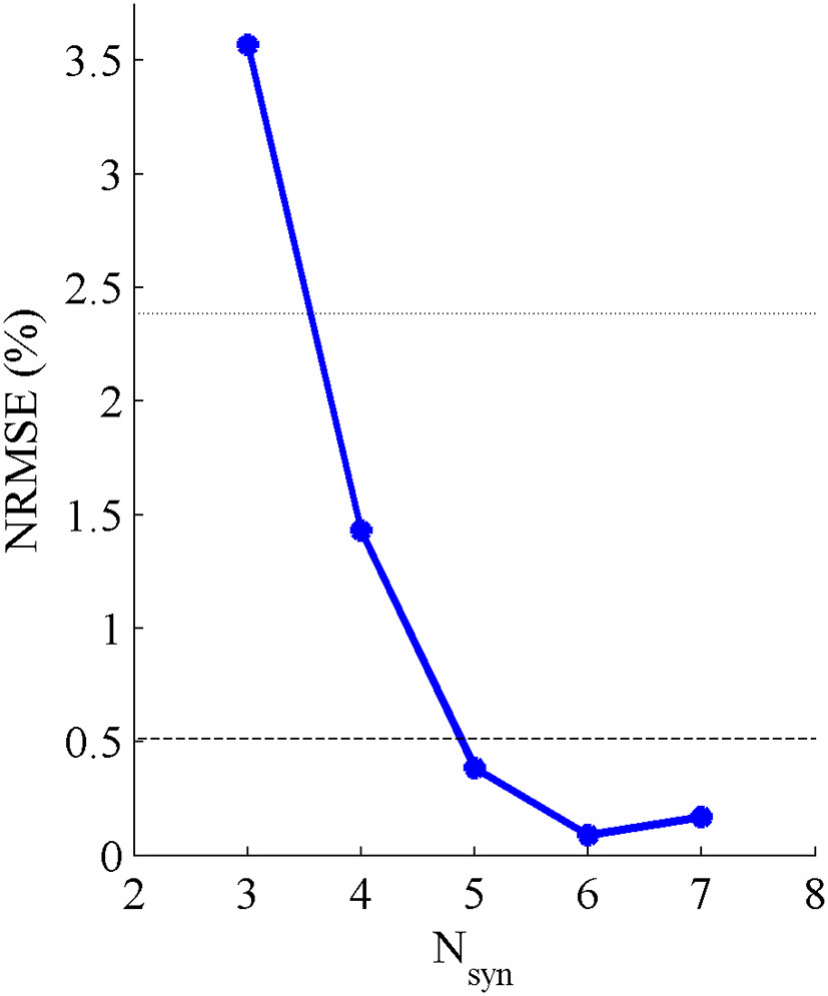
**Variations in *NRMSE* errors between experimental and simulated joint moments with changing values of *N*_*syn*_**. The black dotted line represents the upper limit on the *NRMSE* of 2.38% below which the joint moment errors were considered low, while the black dashed line represents the lower limit of 0.51% below which the risk of a biomechanically redundant synergy set was considered to be high.

**Figure 4:**
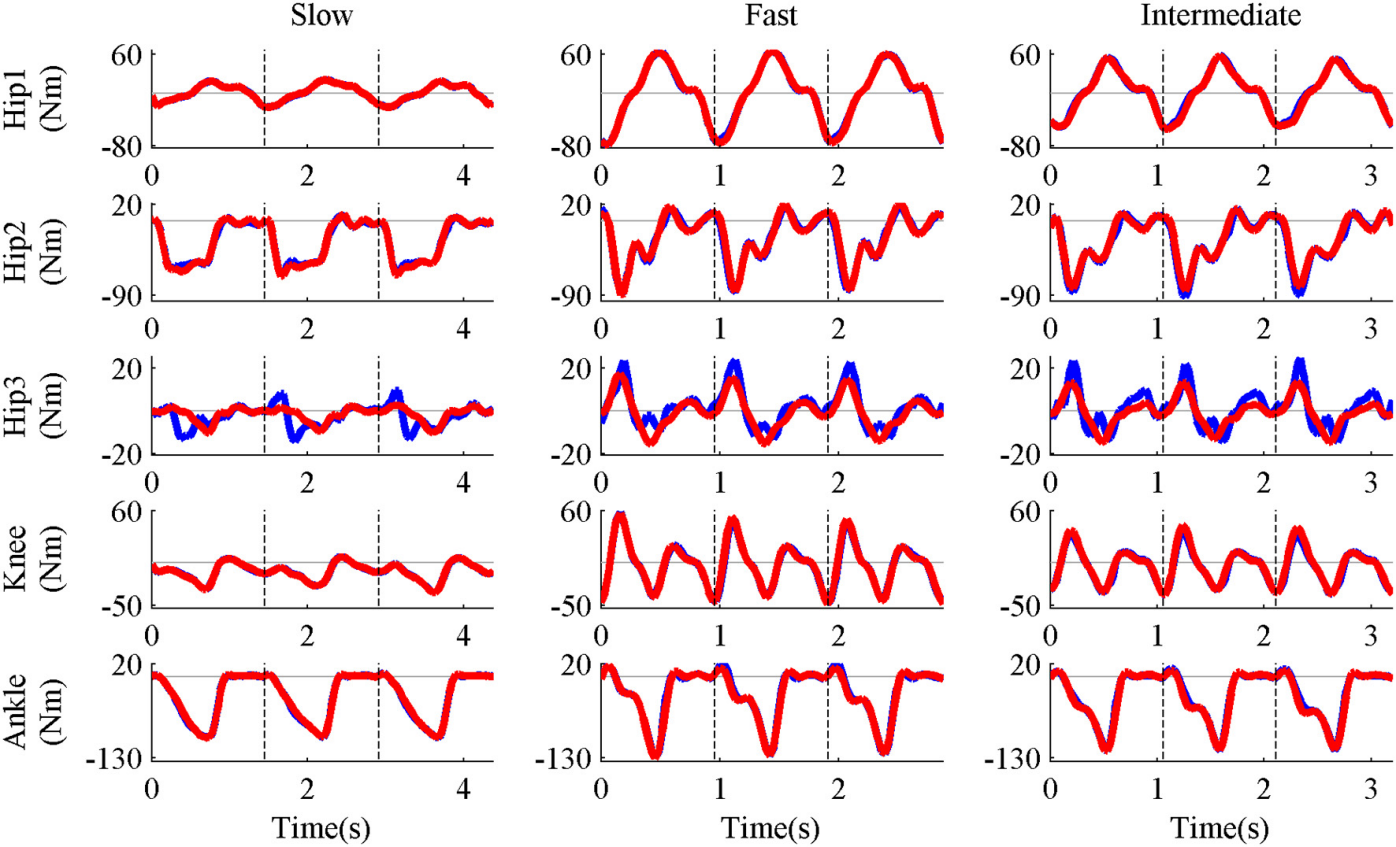
**Representative results of synergy constrained torque decomposition (slow and fast speeds) and the reconstruction of joint moments at the intermediate speed by the pure-synergy solution with *N*_*syn*_ = 4**. The figure presents a comparison of experimental (red line) and simulated (blue line) joint moments over all three gait cycles (separated by the black vertical dashed lines in each plot). The five DOFs of the right leg—hip flexion/extension (Hip1), hip adduction/abduction (Hip2), hip internal/external rotation (Hip3), knee extension/flexion (Knee), and ankle dorsiflexion/plantarflexion (Ankle). With each DOF characterized by bi-directional angular movements (written here as motion1/motion2), it must be noted that positive joint moments were in the direction of motion1 while negative moments were in the direction of motion2.

Five pure-synergy (for *N*_*syn*_ = 3, 4, 5, 6, 7) torque decomposition simulations were successfully completed. The upper and lower limits on the *NRMSE* values were 2.38 and 0.51%, respectively. From a plot of *N*_*syn*_ vs. *NRMSE* (Figure 3), it was seen that only *N*_*syn*_ = 4 exhibited a *NRMSE* value (1.43%) that lay within the upper and lower limits. Hence *N*_*syn*_ = 4 was deemed to be the minimum number of synergies needed to reproduce the biomechanics of the walking task. Figure 4 compares simulated and experimental joint moments from both synergy constrained torque decomposition (*NRMSE* = 1.43%) and the intermediate speed validation test (*NRMSE* = 2.13%) for the case when *N*_*syn*_ = 4. Close similarities between the two sets of moments were observed at all DOFs in Figure 4 with the exception of the hip internal/external rotation where deviations were most notable at the slow walking speed. Joint moments obtained by forward integration of muscle dynamics with excitations generated from the simulated synergy structure showed good agreement with the joint moments from direct collocation (*NRMSE* = 1.78%) thus validating the dynamic consistency of the pure-synergy solution with four muscle synergies.

**Figure 5:**
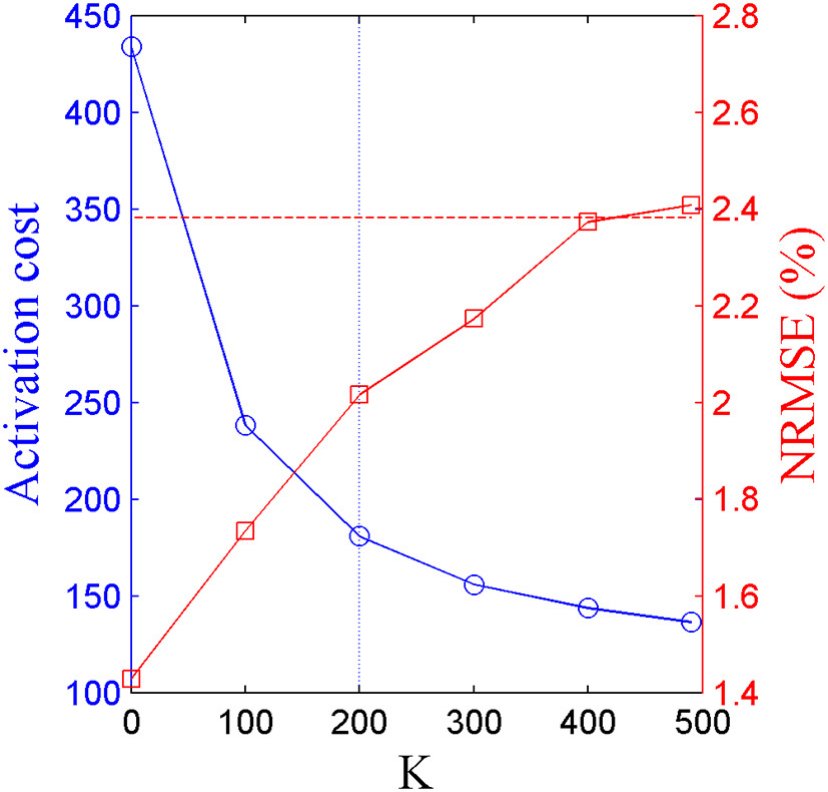
**Trends in activation cost (blue circular markers, left y-axis) and *NRMSE* (red square markers, right y-axis) of optimal-synergy solutions computed with values of *K* ranging from 0 to 500**. The horizontal red dotted line represents the upper limit on *NRMSE* values while the blue dotted vertical line highlights *K* = 200 which satisfied the criterion prescribed by Δ_*K*:*K*+100_.

**Figure 6:**
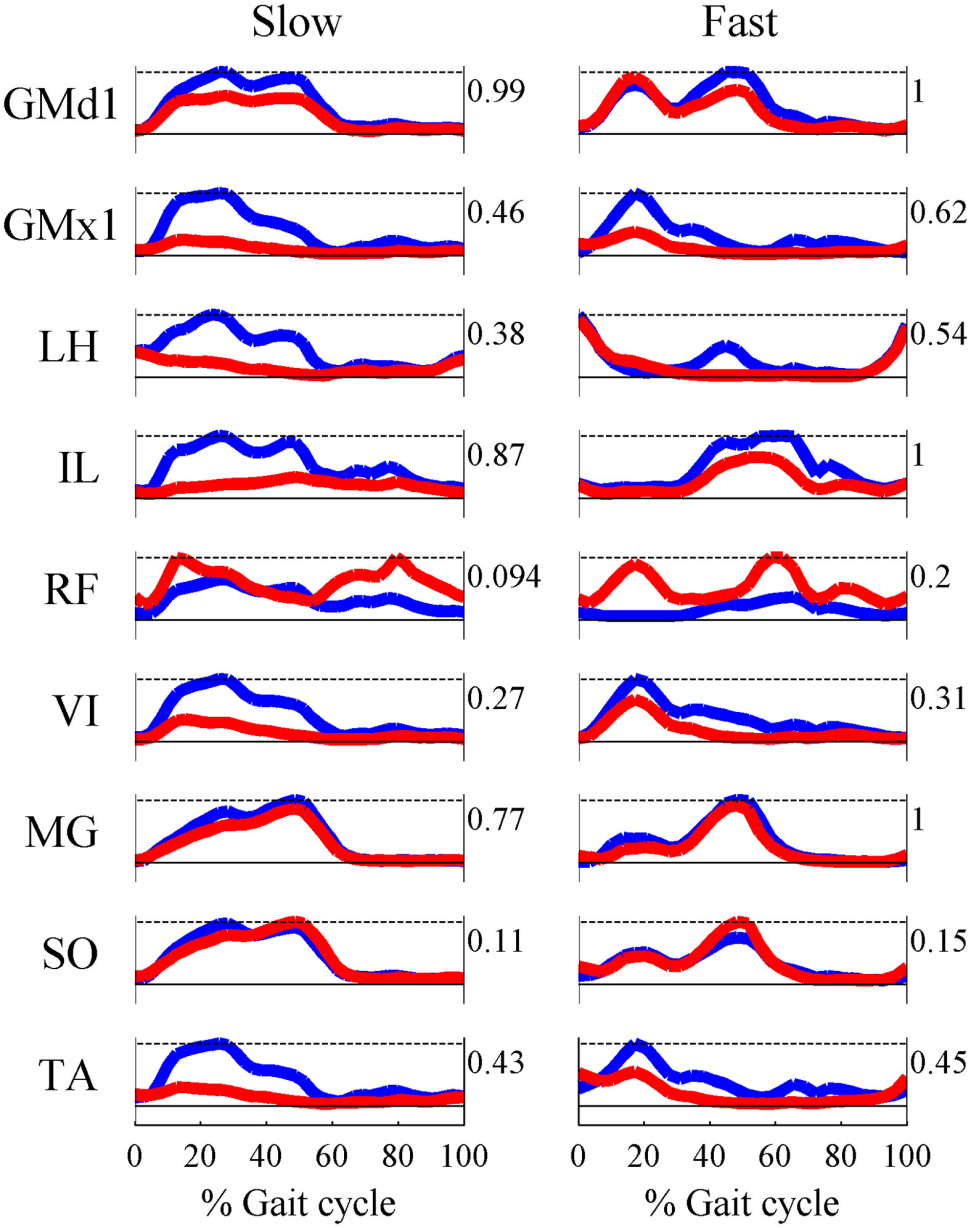
**Comparisons of multiple muscle activation patterns at the slow and fast walking speeds, as computed by the pure-synergy (blue line) and optimal-synergy (red line) solutions**. The numbers to the right of each plot indicate the peak muscle activation level for that muscle.

**Figure 7:**
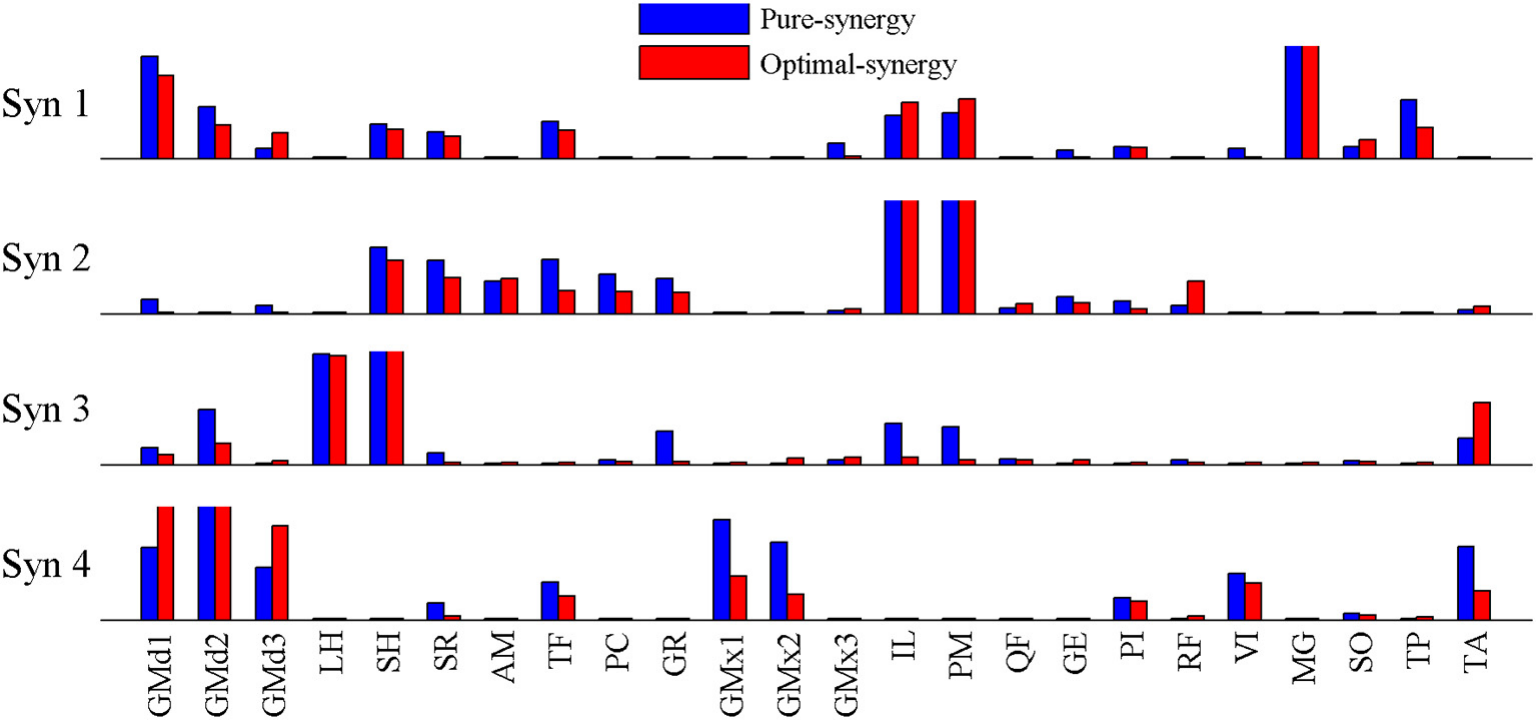
**Comparison of muscle weightings for the 4 synergies computed by the optimal-synergy and pure-synergy solutions**. For the sake of visual representation, weightings in every synergy (both solutions) were scaled such that the highest weighting value was 1. Synergy controls computed by the two solutions are compared in Figure 8A.

**Figure 8:**
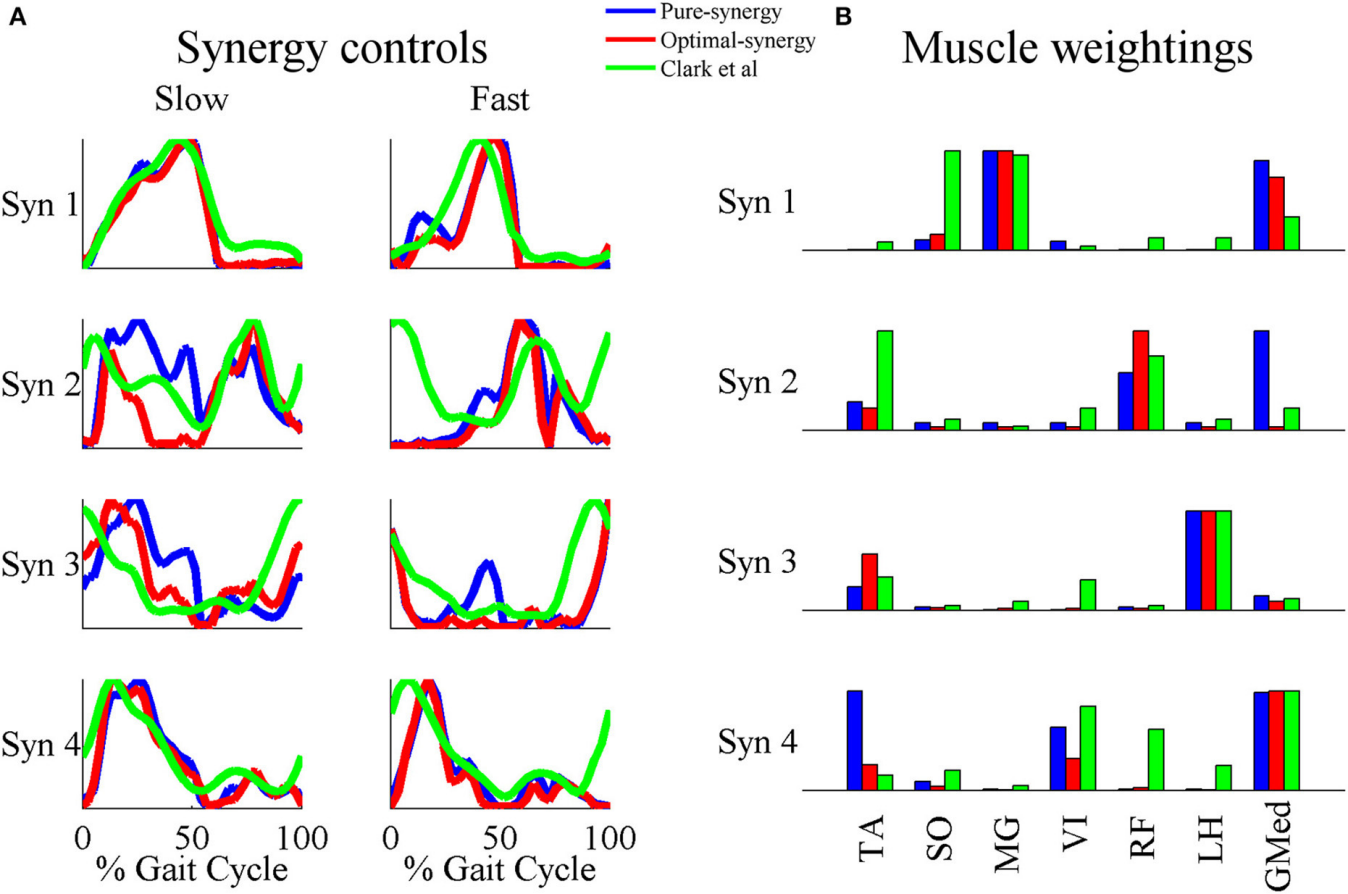
**A comparison of the 4 muscle synergies computed for the pure-synergy and optimal-synergy solutions and those reported by Clark et al. (2010)**. The synergy controls from Clark et al. (2010) reported under the “slow” speed were extracted for walking at 0.6 m/s while the “fast” speed controls were from walking at 1.6 m/s. **(A)** Synergy controls **(B)** Muscle weightings. In panel **(B)**, “LH” stands for lateral hamstring muscle included in Clark et al. which was compared against the LH muscle from the musculoskeletal model in this study. Similarly GMed represents the gluteus medius muscle from Clark et al. and the Gmd1 muscle from the model. For the sake of visual representation in panel **(A)**, all the synergy controls shown were normalized such that the peak magnitude was 1. Similarly, in panel **(B)** weightings in every synergy shown (both simulated solutions and EMG derived) were scaled such that the highest weighting value was 1.

Based on the results for the pure-synergy solution, four synergies were also computed in the optimal-synergy solution. By repeating the simulation for *K* ranging from 0 to 500 (Figure 5) and using the Δ_*K*:*K*+100_ metric defined in Equation (4), the value of *K* was chosen to be 200 and the goodness of joint moment reproduction at this weighting value (*NRMSE* = 2.01%) was also verified. Muscle activation levels predicted by this solution were visibly lower than pure-synergy activations for most muscles in the model (Figure 6). Figure 7 compares muscle weightings from the four synergies computed by the pure-synergy and optimal-synergy solutions (after finding corresponding synergies on the basis of the similarity indices). Synergy-specific similarity indices for weightings in all four synergies in the two solutions (Table 3) were high (>0.8) suggesting largely similar muscle groupings in synergies computed by the two solutions. Figures 8A,B respectively compare synergy controls and weightings from three datasets—optimal-synergy solution, pure-synergy solution, and EMG derived synergies from the study by Clark et al. (2010) which also concluded that four synergies were sufficient for reconstructing experimental EMG patterns for healthy walking at different speeds. Based on values of the calculated similarity indices (Table 3), correspondent synergies were identified and muscle weightings from the optimal-synergy solution were found to be better matched with the experimental ones (mean similarity index *SI*_*os*−*EMG*_ = 0.82 ± 0.09, reported as: mean ± *SD*) as compared to the pure-synergy weightings (mean *SI*_*ps*−*EMG*_ = 0.76 ± 0.14). Thus, muscle groupings in the simulated and EMG derived synergies could be considered to be similar.

**Table 3:**
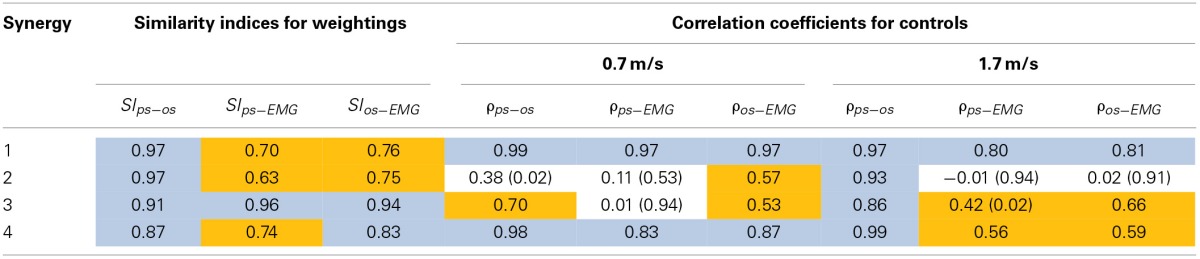
**Quantitative comparisons of synergies from three datasets - pure-synergy solution (ps), optimal-synergy solution (os), and EMG derived synergies (EMG) from Clark et al. (2010)**.

To compare synergies from any two of these three datasets, similarity indices (SI) for muscle weightings and Pearson correlation coefficients (ρ) for synergy controls were computed. When the p-value associated with the correlation was greater than 0.01 (null hypothesis of no correlation), it is reported in parentheses. Other p-values lower than 0.01 indicate high probabilities of the existence of a correlation and have not been explicitly reported in the table. The two datasets which the synergies being compared belonged to are denoted by subscripts; for example *SI*_*ps*−*EMG*_ is the synergy-specific similarity index for weightings from the pure-synergy solution and the EMG synergies. Moderate correlations and similarities (0.8 > ρ, SI ≥ 0.5) are highlighted in orange while good ones (1 > ρ, SI ≥ 0.8) are in blue. Low correlations and similarities (ρ, SI <0.5) have been left uncolored.

**Figure 9:**
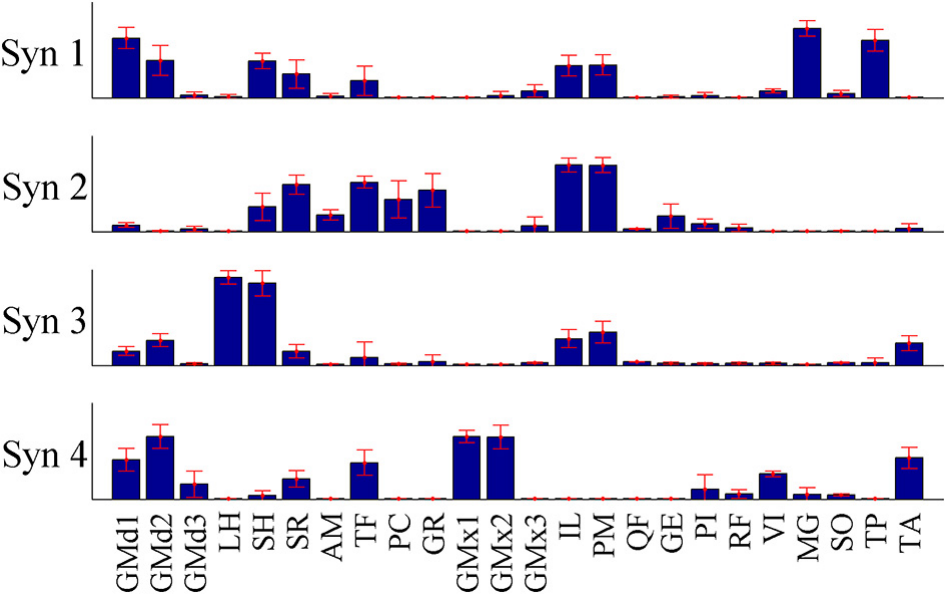
**The mean synergy weightings and standard deviations computed over results from all 15 iterations of the pure-synergy solution with *N*_*syn*_ = 4 (see Appendix)**.

Correlation coefficients were calculated between individual synergy controls from the three datasets and are reported in Table 3. Pure-synergy and optimal-synergy simulations could be considered to yield controls with similar temporal characteristics since they exhibited a high mean correlation (mean ρ_*ps*−*os*_ = 0.86, *p* < 0.01). Synergy controls from the optimal-synergy solution correlated better with the EMG derived synergy controls (mean ρ_*os*−*EMG*_ = 0.60, *p* < 0.01) as compared to the pure-synergy solution (mean ρ_*ps*−*EMG*_ = 0.53, *p* < 0.01). Overall, both optimal-synergy and pure-synergy control signals exhibited moderate mean correlations. At an individual level, the first and fourth synergies showed higher correlations between the simulated and experimental synergy controls while the second synergy showed the least.

## Discussion

The similarities between experimental and simulated joint moments at slow and fast walking speeds (Figure 4) proved the ability of synergy constrained torque decomposition to identify potential muscle synergy groupings that reproduced experimental joint moments in a musculoskeletal model. The forward dynamics test verified that the synergy structures computed by direct collocation were consistent with muscle dynamics. Using the *NRMSE* criterion, four muscle synergies were deemed to be the minimum number of biomechanically relevant synergies needed, which was also supported by the close reconstruction of joint moments for walking at the intermediate speed using four synergies (Figure 4). These joint moments had not been considered during the synergy computation process. The ability of invariant muscle weightings and speed-specific synergy controls to reproduce joint moments in both “training” (slow and fast speeds) and “testing” (intermediate speed) data which exhibited variations in the gait cycle time-periods and joint moment profiles highlights the potential of such a controller layout for actuating healthy walking movements. The deviations in hip internal/external rotation joint moment errors (Figure 4) could be attributed to the lack of well-directed muscle action about that DOF in the presence of synergy groupings since the gait2354 musculoskeletal model used in this study was a simplified version of the “gait2392” model (containing 92 muscles and also distributed with OpenSim), with a number of muscles removed for computational efficiency. Even though synergy constrained torque decomposition could reproduce the biomechanical outputs defined in this study (joint moments), users of this technique need to verify the accuracy of reproduction when a different output is of interest (e.g., joint powers).

The *NRMSE* criterion used to choose an appropriate number of synergies gave us four synergies which was consistent with the observations of Clark et al. (2010). This similarity was worth noting because EMG from only eight muscles were recorded in that study, as opposed to the 24 muscles in the musculoskeletal model used in this one. A study by Steele et al. (2013) observed that while the structure of muscle weightings computed by EMG factorization depends on which muscles EMG is recorded from, the dependency can be reduced if EMG from dominant muscles (high maximum isometric forces) are always included. The eight muscles in the study by Clark et al. (2010) were also dominant muscles in the musculoskeletal model used in this study, which might explain why simulated and EMG derived synergies were similar despite the different number of muscles in the two studies.

Some other studies have reported five EMG derived synergies (Ivanenko et al., 2004; Cappellini et al., 2006) for locomotion tasks which would have been considered redundant on the basis of the *NRMSE* criterion used in this study. It is possible that the minimum number of synergies might increase when decomposing joint moments from more diverse walking conditions (e.g., more speeds, various floor inclinations, etc.). With a small family of movements, methods such as torque decomposition and NNMF would only identify a single merged synergy whose muscle weightings would be an algebraic sum of the two individual sets of weightings. To test for the presence of these potentially “merged” synergies, an increase in the size of that family of movements would thus be necessary.

If joint moments from each DOF were collectively represented in a five dimensional vector, the vector space spanned by the instantaneous joint moments during a family of movements tasks might be of a lower dimension (≤5), which is termed the “dimensionality” of the joint moments. A recent study by Russo et al. (2014) investigated relationships between the dimensionality of joint moments and muscle activity (the number of EMG derived muscle synergies) during reaching tasks. Muscle dimensionality had to theoretically be at least one higher than the joint moment dimensionality since muscles only produce forces in tension (i.e., non-negative excitations). However, they observed the number of synergies to usually be higher than this minimum, which was potentially necessitated by the non-linear, history-dependent muscle behavior and additional performance requirements such as minimization of muscle effort. Following the procedures of Russo et al. (2014), a principal component analysis applied to joint moments for slow and fast walking in this study, revealed the need for three principal components to account for at least 90% of the variance in the data.

This implied the need for a minimum of four muscle synergies, which concurred with the pure-synergy simulation despite the latter considering non-linear muscle behavior. It is therefore possible that the modeled non-linearities did not significantly constrain muscle force production during walking, although this would require further investigation with multiple subjects in order to be generalizable. The optimal-synergy solution demonstrated that the same dimensionality of four synergies could also reduce muscular effort in comparison to the pure-synergy solution (Figure 6), although a higher number of optimal-synergies could possibly have reduced muscle effort even further. This is worth further investigation since the results will reveal whether optimality needs necessarily influence the minimum number of muscle synergies for human walking. Since four synergies only possessed the minimum dimensionality required, it was possible that this reduced the size of feasible muscle grouping schemes for reproducing experimental joint moments in the model. This could potentially explain the similar muscle groupings in pure-synergy and optimal-synergy solutions.

Direct comparison of muscle weightings and synergy control magnitudes from the two simulated solutions could be inappropriate, since weightings and controls can theoretically vary between zero and infinity while only excitations resulting from their linear combination (Equation 1E) are required to lie between 0 and 1. While different simulations may compute synergies which satisfy that constraint, the weightings and controls themselves may have very different magnitudes. Such direct comparisons of magnitude must therefore be reserved for muscle excitations or activations. However, values of similarity indices comparing muscle weightings suggested that the same muscles were weighted heavily within corresponding synergies—implying identical “muscle groupings” within synergies in the optimal-synergy and pure-synergy solutions. Based on the computed correlation coefficients, the temporal variations in synergy controls from the pure and optimal-synergy solutions were also similar.

Despite control signals having similar timing characteristics, similar muscle groupings, and reproducing the same joint moment patterns, muscle activations were lower in the optimal-synergy solution (Figure 6). The pure-synergy and optimal-synergy solutions differed only with regards to the additional goal of minimizing muscle activations in the latter. Thus, introducing the notion of a muscle grouping scheme and fixing the number of groups in that scheme to the minimum necessary for healthy human gait, seemed to automatically direct the system toward one muscle grouping scheme and a concomitant set of control signal temporal profiles for each group irrespective of the optimality requirement. Given the anatomy of the human lower limb and the mechanical requirements for gait, synergies could be a useful strategy for achieving optimized control of walking by reducing the dimensionality of the problem. How closely the activations from an optimal-synergy solution resemble those from a purely optimal solution (in the absence of any synergy constraints) still remains to be investigated.

The similarities between simulated and EMG derived synergies (Figure 8)—especially muscle weightings and also between multiple pairs of synergy controls (Table 3)—despite being derived from different experimental measurements is another finding from this study. Closer agreement between optimal-synergy and experimentally derived synergies (with regards to both weightings and controls) was similar to the observations of Borzelli et al. (2013) who noted that minimizing the magnitudes of synergy controls as opposed to individual muscle activations better agreed with experimental muscle activations from isometric hand-force generation. The rationale behind computing synergies from EMG is that the presence of a synergy based controller must manifest as a structured variation in the control signals reaching individual muscles for a family of movements. On similar lines, the synergy constrained torque decomposition approach hypothesized that the presence of muscle synergies would be reflected in variations of movement kinematics and kinetics. The similarities between experimental and simulated synergies suggest that both variations in EMG and movement biomechanics point toward the presence of similar underlying synergy based control structures for healthy human walking. The comparison of simulated walking synergies with EMG derived ones from Clark et al. (2010) however came with an implicit assumption that EMG derived synergies showed similarities in structure across subjects from a healthy population. Such inter-subject similarities have been observed by earlier studies (Ivanenko et al., 2004, 2005; De Groote et al., 2014), and even if the differences in simulated synergy structures with respect to Clark et al. (2010) potentially lay within the envelope of the limited inter-subject variability, it would not be easy to ascertain.

While the controller layout hypothesized by this study involves invariant muscle weightings and task-specific synergy controls collectively actuating a family of movements, other controller layouts have been proposed in prior literature. These alternative layouts include temporal invariance (Ivanenko et al., 2004; Cappellini et al., 2006) where synergy controls are invariant and muscle weightings are movement-specific, and spatio-temporal invariance (d'Avella et al., 2006) where a synergy consists of invariant, muscle-specific, fixed duration signals whose onset times and scaling factors are modulated to produce different movements. All three proposed layouts however concur on the assumption of a linear synergy combination law. For human walking in particular, factor analysis of EMG has revealed the potential of a temporal invariance (after the time-scale has been normalized to gait cycle duration) model for actuating families of walking movements (Lacquaniti et al., 2012) such as at various speeds (Ivanenko et al., 2004), and forward/backward gait (Ivanenko et al., 2008). While the results of the undertaken study suggest that a spatially invariant synergy structure is capable of actuating gait over different speeds, the OCP in Equation (1) can be modified in a future investigation to have invariant control signals and movement-specific weightings, thus testing the biomechanical feasibility of the temporal invariance scheme for gait.

Synergy constrained torque decomposition permits the use of experimental motion capture data as an alternative to EMG, for deducing the structure of muscle synergies. Additionally, synergies deduced from joint moments are quantitatively related to the model's biomechanical output which permits further analyses such as sensitivity studies. The ability to assign synergy groupings to all muscles in the model, unambiguously defines a system-level synergy control strategy whose biomechanical relevance could be tested. Despite such advantages, synergy constrained torque decomposition is still susceptible to errors due to noisy marker data caused by skin motion, the uncertainties of inverse dynamics (Challis and Kerwin, 1996)—approximated limb lengths, inertial parameters, and errors in force/center of pressure measurement (Camargo-Junior et al., 2013)—approximations of the geometry and dynamic models used to represent muscles and the limitations of determining those dynamic model parameters on a subject-specific basis. Computational times for synergy constrained torque decomposition depended on factors such as the initial guess and the weighting constant value for the optimal-synergy solution and ranged between 10 and 20 min. This was relatively much slower compared to NNMF of EMG which converges in the order of a few seconds. Thus, if similarities between EMG derived and joint moment based synergies could be generalized to other subjects and activities, the former method could serve as a rapid synergy assessment tool, while the latter could be reserved for detailed investigations.

Multiple prior studies have used dynamic musculoskeletal simulations to study synergy based control of movements (Berniker et al., 2009; Kargo et al., 2010; Ruckert and d'Avella, 2013). With regards to gait, Neptune et al. (2009) studied the contributions of four EMG derived synergies toward sub-tasks such as forward propulsion and weight acceptance during health, sagittal plane walking. This was then extended to study how synergies adapt to changes in body-weight (McGowan et al., 2010) and investigations of synergy contributions to sub-tasks during 3D walking (Allen and Neptune, 2012). Sartori et al. (2013) demonstrated how EMG derived synergies could reconstruct joint moments for a variety of locomotive tasks. This study motivates an alternative application of musculoskeletal models in the direct computation of muscle synergies capable of producing the desired biomechanical output.

In comparison to prior simulation methods which deduced muscle synergies in either static kinematic configurations or through quasi-static approximations of dynamic tasks, the methodology in this study accounted for the non-linear, history-dependent muscle dynamics which could influence synergy structure. The addition of dynamic, afferent feedback models in the OCP will permit future studies to investigate the voluntary and feedback based components of muscle synergy structure. For instance, such an approach could be used to address the question outlined by Kutch and Valero-Cuevas (2012) regarding the possibilities of feedback-induced muscle excitation signals being mistaken for muscle synergies of neural origin, in the context of dynamic movement tasks. In comparison to previous approaches computing muscle synergies through NNMF of optimal activations (Steele et al., 2013; De Groote et al., 2014), this study proposed a method where synergies could be investigated independent of any optimality assumptions (pure-synergy solution) which could be optionally included (optimal-synergy solution). With the NNMF approach, there is also a risk that small errors in muscle activations reconstructed from the synergies might correspond to large errors in the biomechanical output (in this study, joint moments) of a feed-forward model driven by the reconstructed activations, due to non-linear model behaviors. This study averts this possibility by directly incorporating the muscle synergy hypothesis in the dynamic simulation process.

A limitation of this investigation is the application of the method to results from only one subject. While this small dataset served the purpose of demonstrating the formulation and potential applications of synergy constrained torque decomposition, the similarities of the computed synergies to EMG derived ones and the relationships between optimal-synergy and pure-synergy solutions deserves further investigation using more subjects and a larger family of movements. Nevertheless, muscle synergies deduced from six gait cycles of variable joint moment data still demonstrated biomechanical relevance by reproducing joint moments from three unseen gait cycles at an intermediate speed. This study also used a simplified musculoskeletal model for reducing the simulation's computation time, which could have potentially led to the deviations observed in the hip internal/external rotation moment. Since direct collocation was solved by a gradient based optimization algorithm, there was the risk that the solution only locally minimized the cost function. While such a risk is characteristic of any method using gradient based optimization (including NNMF), it was verified that different, arbitrary initial guesses yielded similar muscle grouping schemes (see Appendix). Given the range of possibilities for the arbitrary initial guesses, untested initial guesses yielding a different synergy structure could still potentially exist.

In summary, this study presents a novel technique for decomposing joint moments into underlying muscle synergies. Similarities in the simulated and EMG derived synergy structures from Clark et al. (2010) suggested that analyzing both types of experimental data reveals similar underlying control structures. Muscle synergies from the pure-synergy and optimal-synergy solutions exhibited similar muscle groupings and temporal control signal features. Future investigations using the torque decomposition approach could investigate whether more detailed models of the musculoskeletal system with finer muscle discretization affect the results we have observed by representing muscle mechanical functions more accurately. Computing synergies from joint moments for a more diverse family of locomotion movements would also aid in determining the exact range of the family of movements which a given synergy set can actuate.

### Conflict of interest statement

The authors declare that the research was conducted in the absence of any commercial or financial relationships that could be construed as a potential conflict of interest.

## Appendix

### How the initial guess influences muscle groupings of the pure-synergy solution

The initial guess for the pure-synergy solution was obtained by first assigning arbitrary muscle excitation patterns to the 24 muscles, over the duration of the three gait cycles at slow and fast walking speeds. These arbitrary excitations consisted of either (a) constant excitation levels over the entire time duration or (b) constant excitations interspersed with sinusoidal pulses. The number of muscles receiving both categories of excitation patterns and the selection of muscles belonging to each category were based on randomly generated integers (“randi” function in MATLAB). The magnitude of the constant excitation levels and the amplitude and timing of the sinusoidal pulses were also randomly assigned (“rand” function in MATLAB). Non-negative matrix factorization (NNMF) was used to decompose these arbitrary excitations into muscle weightings and synergy controls. The pure-synergy solution was repeated using 15 different initial guesses with *N*_*syn*_ set to a value of 4 synergies. For each such solution, muscle weightings from the four synergies were treated as vectors that were normalized to have a magnitude of 1. This justified the computation of the mean and standard deviation of the muscle weightings from the 15 solutions, which is presented in Figure 9.

For each synergy, the similarity index (described in Section “Simulations”) was used to quantify the similarity between the mean muscle weighting vector, and the vector from each of the 15 solutions. The mean similarity index (average of the 15 indices) was greater than 0.95 for each of the four synergies. Since these similarity indices were greater than 0.8 (interpretation of similarity index values is discussed in Section “Simulations”), they were considered to indicate highly similar muscle groupings in the muscle synergies from the 15 pure synergy solutions. The solutions resulted in NRMSE values in the range of 1.43–1.58% and the solution with the lowest NRMSE was taken to be the final pure-synergy solution.

Muscle synergies in this study were computed using a gradient-based numerical optimization scheme. In such a scheme there is theoretically always a solution which results in a lower cost function value compared to the current solution even if only infinitesimal improvements are made, and the optimization terminates when certain tolerances have been met. It is possible that a certain muscle grouping scheme was more successful at numerically minimizing the joint moment errors to suit the required tolerances (which were invariant between repetitions of the pure-synergy simulation), resulting in multiple solutions arriving upon the same muscle grouping scheme. As mentioned in the discussion, 4 synergies were the minimum required to explain the dimensionality of the walking joint moments used in this study. It is likely that this further restricted the set of feasible pure-synergy muscle groupings. Nevertheless, as with all gradient-based optimization approaches (including NNMF), there was a risk that only a local minima was attained during all 15 pure-synergy solutions and a different scheme for initializing the direct collocation scheme might yield a solution with a significantly different grouping scheme. The need for an invariant set of muscle weightings to reproduce joint moments from two walking speeds which exhibited differences in magnitude and temporal profiles, might have also constrained the number of feasible muscle grouping schemes.

## References

[B1] Ackermann M. Van Den Bogert A. J. (2010). Optimality principles for model-based prediction of human gait. J. Biomech. 43, 1055–1060. 10.1016/j.jbiomech.2009.12.012 20074736 PMC2849893

[B2] Allen J. L. Neptune R. R. (2012). Three-dimensional modular control of human walking. J. Biomech. 45, 2157–2163. 10.1016/j.jbiomech.2012.05.037 22727468 PMC3405171

[B3] Anderson F. C. Pandy M. G. (1999). A dynamic optimization solution for vertical jumping in three dimensions. Comput. Methods Biomech. Biomed. Eng. 2, 201–231. 10.1080/10255849908907988 11264828

[B4] Arnold E. M. Hamner S. R. Seth A. Millard M. Delp S. L. (2013). How muscle fiber lengths and velocities affect muscle force generation as humans walk and run at different speeds. J. Exp. Biol. 216, 2150–2160. 10.1242/jeb.075697 23470656 PMC3656509

[B5] Berniker M. Jarc A. Bizzi E. Tresch M. C. (2009). Simplified and effective motor control based on muscle synergies to exploit musculoskeletal dynamics. Proc. Natl. Acad. Sci. U.S.A. 106, 7601–7606. 10.1073/pnas.0901512106 19380738 PMC2678607

[B6] Borzelli D. Berger D. J. Pai D. K. d'Avella A. (2013). Effort minimization and synergistic muscle recruitment for three-dimensional force generation. Front. Comput. Neurosci. 7:186. 10.3389/fncom.2013.00186 24391581 PMC3868911

[B7] Buchanan T. S. Lloyd D. G. Manal K. Besier T. F. (2005). Estimation of muscle forces and joint moments using a forward-inverse dynamics model. Med. Sci. Sports Exerc. 37, 1911–1916. 10.1249/01.mss.0000176684.24008.6f 16286861

[B8] Camargo-Junior F. Ackermann M. Loss J. F. Sacco I. C. (2013). Influence of center of pressure estimation errors on 3D inverse dynamics solutions during gait at different velocities. J. Appl. Biomech. 29, 790–797. Available online at: http://journals.humankinetics.com/jab-back-issues/jab-volume-29-issue-6-december/influence-of-center-of-pressure-estimation-errors-on-3-d-inverse-dynamics-solutions-during-gait-at-different-velocities 23343751 10.1123/jab.29.6.790

[B9] Cappellini G. Ivanenko Y. P. Poppele R. E. Lacquaniti F. (2006). Motor patterns in human walking and running. J. Neurophysiol. 95, 3426–3437. 10.1152/jn.00081.2006 16554517

[B10] Challis J. H. Kerwin D. G. (1996). Quantification of the uncertainties in resultant joint moments computed in a dynamic activity. J. Sports Sci. 14, 219–231. 10.1080/02640419608727706 8809714

[B11] Cheung V. C. d'Avella A. Tresch M. C. Bizzi E. (2005). Central and sensory contributions to the activation and organization of muscle synergies during natural motor behaviors. J. Neurosci. 25, 6419–6434. 10.1523/JNEUROSCI.4904-04.2005 16000633 PMC6725265

[B12] Chvatal S. A. Ting L. H. (2013). Common muscle synergies for balance and walking. Front. Comput. Neurosci. 7:48. 10.3389/fncom.2013.00048 23653605 PMC3641709

[B13] Clark D. J. Ting L. H. Zajac F. E. Neptune R. R. Kautz S. A. (2010). Merging of healthy motor modules predicts reduced locomotor performance and muscle coordination complexity post-stroke. J. Neurophysiol. 103, 844–857. 10.1152/jn.00825.2009 20007501 PMC2822696

[B14] Crowninshield R. D. Brand R. A. (1981). A physiologically based criterion of muscle force prediction in locomotion. J. Biomech. 14, 793–801. 10.1016/0021-9290(81)90035-X 7334039

[B15] d'Avella A. Bizzi E. (2005). Shared and specific muscle synergies in natural motor behaviors. Proc. Natl. Acad. Sci. U.S.A. 102, 3076–3081. 10.1073/pnas.0500199102 15708969 PMC549495

[B16] d'Avella A. Portone A. Fernandez L. Lacquaniti F. (2006). Control of fast-reaching movements by muscle synergy combinations. J. Neurosci. 26, 7791–7810. 10.1523/JNEUROSCI.0830-06.2006 16870725 PMC6674215

[B17] d'Avella A. Saltiel P. Bizzi E. (2003). Combinations of muscle synergies in the construction of a natural motor behavior. Nat. Neurosci. 6, 300–308. 10.1038/nn1010 12563264

[B18] De Groote F. Jonkers I. Duysens J. (2014). Task constraints and minimization of muscle effort result in a small number of muscle synergies during gait. Front. Comput. Neurosci. 8:115. 10.3389/fncom.2014.00115 25278871 PMC4167006

[B19] Delp S. L. Anderson F. C. Arnold A. S. Loan P. Habib A. John C. T.. (2007). OpenSim: open-source software to create and analyze dynamic simulations of movement. IEEE Trans. Biomed. Eng. 54, 1940–1950. 10.1109/TBME.2007.901024 18018689

[B20] Delp S. L. Loan J. P. Hoy M. G. Zajac F. E. Topp E. L. Rosen J. M. (1990). An interactive graphics-based model of the lower extremity to study orthopaedic surgical procedures. IEEE Trans. Biomed. Eng. 37, 757–767. 10.1109/10.102791 2210784

[B21] De Luca C. J. (1997). The use of surface electromyography in bio-mechanics. J. Appl. Biomech. 13, 135–163.

[B22] De Rugy A. Loeb G. E. Carroll T. J. (2013). Are muscle synergies useful for neural control? Front. Comput. Neurosci. 7:19. 10.3389/fncom.2013.00019 23519326 PMC3604633

[B23] Disselhorst-Klug C. Schmitz-Rode T. Rau G. (2009). Surface electromyography and muscle force: limits in sEMG-force relationship and new approaches for applications. Clin. Biomech. (Bristol, Avon) 24, 225–235. 10.1016/j.clinbiomech.2008.08.003 18849097

[B24] Farris D. J. Sawicki G. S. (2012). Human medial gastrocnemius force-velocity behavior shifts with locomotion speed and gait. Proc. Natl. Acad. Sci. U.S.A. 109, 977–982. 10.1073/pnas.1107972109 22219360 PMC3271879

[B25] Gill P. E. Murray W. Saunders M. A. (2002). SNOPT: an SQP algorithm for large-scale constrained optimization. SIAM J. Optim. 12, 979–1006 10.1137/S1052623499350013

[B26] Giszter S. F. Mussa-Ivaldi F. A. Bizzi E. (1993). Convergent force fields organized in the frog's spinal cord. J. Neurosci. 13, 467–491. 8426224 10.1523/JNEUROSCI.13-02-00467.1993 PMC6576636

[B27] He J. Levine W. S. Loeb G. E. (1991). Feedback gains for correcting small perturbations to standing posture. IEEE Trans. Autom. Control 36, 322–332 10.1109/9.73565

[B28] Ivanenko Y. P. Cappellini G. Dominici N. Poppele R. E. Lacquaniti F. (2005). Coordination of locomotion with voluntary movements in humans. J. Neurosci. 25, 7238–7253. 10.1523/JNEUROSCI.1327-05.2005 16079406 PMC6725226

[B29] Ivanenko Y. P. Cappellini G. Poppele R. E. Lacquaniti F. (2008). Spatiotemporal organization of alpha-motoneuron activity in the human spinal cord during different gaits and gait transitions. Eur. J. Neurosci. 27, 3351–3368. 10.1111/j.1460-9568.2008.06289.x 18598271

[B30] Ivanenko Y. P. Poppele R. E. Lacquaniti F. (2004). Five basic muscle activation patterns account for muscle activity during human locomotion. J. Physiol. 556, 267–282. 10.1113/jphysiol.2003.057174 14724214 PMC1664897

[B31] Ivanenko Y. P. Poppele R. E. Lacquaniti F. (2006). Motor control programs and walking. Neuroscientist 12, 339–348. 10.1177/1073858406287987 16840710

[B32] Jacobs R. Macpherson J. M. (1996). Two functional muscle groupings during postural equilibrium tasks in standing cats. J. Neurophysiol. 76, 2402–2411. 8899613 10.1152/jn.1996.76.4.2402

[B33] John C. T. Anderson F. C. Higginson J. S. Delp S. L. (2013). Stabilisation of walking by intrinsic muscle properties revealed in a three-dimensional muscle-driven simulation. Comput. Methods Biomech. Biomed. Eng. 16, 451–462. 10.1080/10255842.2011.627560 22224406 PMC3397280

[B34] Kaplan M. L. Heegaard J. H. (2001). Predictive algorithms for neuromuscular control of human locomotion. J. Biomech. 34, 1077–1083. 10.1016/S0021-9290(01)00057-4 11448699

[B35] Kargo W. J. Ramakrishnan A. Hart C. B. Rome L. C. Giszter S. F. (2010). A simple experimentally based model using proprioceptive regulation of motor primitives captures adjusted trajectory formation in spinal frogs. J. Neurophysiol. 103, 573–590. 10.1152/jn.01054.2007 19657082 PMC2807239

[B36] Kistemaker D. A. Van Soest A. J. Wong J. D. Kurtzer I. Gribble P. L. (2013). Control of position and movement is simplified by combined muscle spindle and Golgi tendon organ feedback. J. Neurophysiol. 109, 1126–1139. 10.1152/jn.00751.2012 23100138 PMC3569141

[B37] Kutch J. J. Valero-Cuevas F. J. (2012). Challenges and new approaches to proving the existence of muscle synergies of neural origin. PLoS Comput. Biol. 8:e1002434. 10.1371/journal.pcbi.1002434 22570602 PMC3342930

[B38] Lacquaniti F. Ivanenko Y. P. Zago M. (2012). Patterned control of human locomotion. J. Physiol. 590, 2189–2199. 10.1113/jphysiol.2011.215137 22411012 PMC3424743

[B39] Lee D. D. Seung H. S. (1999). Learning the parts of objects by non-negative matrix factorization. Nature 401, 788–791. 10.1038/44565 10548103

[B40] Lu T. W. O'Connor J. J. (1999). Bone position estimation from skin marker co-ordinates using global optimisation with joint constraints. J. Biomech. 32, 129–134. 10.1016/S0021-9290(98)00158-4 10052917

[B41] McGowan C. P. Neptune R. R. Clark D. J. Kautz S. A. (2010). Modular control of human walking: adaptations to altered mechanical demands. J. Biomech. 43, 412–419. 10.1016/j.jbiomech.2009.10.009 19879583 PMC2813323

[B42] McKay J. L. Ting L. H. (2012). Optimization of muscle activity for task-level goals predicts complex changes in limb forces across biomechanical contexts. PLoS Comput. Biol. 8:e1002465. 10.1371/journal.pcbi.1002465 22511857 PMC3325175

[B43] Mesin L. Merletti R. Rainoldi A. (2009). Surface EMG: the issue of electrode location. J. Electromyogr. Kinesiol. 19, 719–726. 10.1016/j.jelekin.2008.07.006 18829347

[B44] Mileusnic M. P. Brown I. E. Lan N. Loeb G. E. (2006). Mathematical models of proprioceptors. I. Control and transduction in the muscle spindle. J. Neurophysiol. 96, 1772–1788. 10.1152/jn.00868.2005 16672301

[B45] Mileusnic M. P. Loeb G. E. (2006). Mathematical models of proprioceptors. II. Structure and function of the Golgi tendon organ. J. Neurophysiol. 96, 1789–1802. 10.1152/jn.00869.2005 16672300

[B46] Modenese L. Gopalakrishnan A. Phillips A. T. (2013). Application of a falsification strategy to a musculoskeletal model of the lower limb and accuracy of the predicted hip contact force vector. J. Biomech. 46, 1193–1200. 10.1016/j.jbiomech.2012.11.045 23427941

[B47] Moghadam M. N. Aminian K. Asghari M. Parnianpour M. (2011). How well do the muscular synergies extracted via non-negative matrix factorisation explain the variation of torque at shoulder joint? Comput. Methods Biomech. Biomed. Engin. 16, 291–301. 10.1080/10255842.2011.617705 21970618

[B48] Neptune R. R. Clark D. J. Kautz S. A. (2009). Modular control of human walking: a simulation study. J. Biomech. 42, 1282–1287. 10.1016/j.jbiomech.2009.03.009 19394023 PMC2696580

[B49] Perotto A. Delagi E. F. Ebooks Corporation. (2011). Anatomical Guide for the Electromyographer the Limbs and Trunk, 5th Edn. Springfield, IL: Charles C. Thomas Publisher.

[B50] Ruckert E. d'Avella A. (2013). Learned parametrized dynamic movement primitives with shared synergies for controlling robotic and musculoskeletal systems. Front. Comput. Neurosci. 7:138. 10.3389/fncom.2013.00138 24146647 PMC3797962

[B51] Russo M. D'andola M. Portone A. Lacquaniti F. d'Avella A. (2014). Dimensionality of joint torques and muscle patterns for reaching. Front. Comput. Neurosci. 8:24. 10.3389/fncom.2014.00024 24624078 PMC3939605

[B52] Sartori M. Gizzi L. Lloyd D. G. Farina D. (2013). A musculoskeletal model of human locomotion driven by a low dimensional set of impulsive excitation primitives. Front. Comput. Neurosci. 7:79. 10.3389/fncom.2013.00079 23805099 PMC3693080

[B53] Singh T. Varadhan S. K. Zatsiorsky V. M. Latash M. L. (2010). Fatigue and motor redundancy: adaptive increase in finger force variance in multi-finger tasks. J. Neurophysiol. 103, 2990–3000. 10.1152/jn.00077.2010 20357060 PMC2888234

[B54] Steele K. M. Tresch M. C. Perreault E. J. (2013). The number and choice of muscles impact the results of muscle synergy analyses. Front. Comput. Neurosci. 7:105. 10.3389/fncom.2013.00105 23964232 PMC3737463

[B55] Thelen D. G. (2003). Adjustment of muscle mechanics model parameters to simulate dynamic contractions in older adults. J. Biomech. Eng. 125, 70–77. 10.1115/1.1531112 12661198

[B56] Thelen D. G. Anderson F. C. (2006). Using computed muscle control to generate forward dynamic simulations of human walking from experimental data. J. Biomech. 39, 1107–1115. 10.1016/j.jbiomech.2005.02.010 16023125

[B57] Ting L. H. Macpherson J. M. (2005). A limited set of muscle synergies for force control during a postural task. J. Neurophysiol. 93, 609–613. 10.1152/jn.00681.2004 15342720

[B58] Tresch M. C. Cheung V. C. d'Avella A. (2006). Matrix factorization algorithms for the identification of muscle synergies: evaluation on simulated and experimental data sets. J. Neurophysiol. 95, 2199–2212. 10.1152/jn.00222.2005 16394079

[B59] Tresch M. C. Saltiel P. Bizzi E. (1999). The construction of movement by the spinal cord. Nat. Neurosci. 2, 162–167. 10.1038/5721 10195201

[B60] Walter J. P. Kinney A. L. Banks S. A. d'Lima D. D. Besier T. F. Lloyd D. G.. (2014). Muscle synergies may improve optimization prediction of knee contact forces during walking. J. Biomech. Eng. 136:021031. 10.1115/1.4026428 24402438 PMC5101026

[B61] Zajac F. E. Gordon M. E. (1989). Determining muscle's force and action in multi-articular movement. Exerc. Sport Sci. Rev. 17, 187–230. 2676547

